# Differences in fatty infiltration in thigh muscles and physical function between people with and without knee osteoarthritis and similar body mass index: a cross-sectional study

**DOI:** 10.1186/s12891-025-08347-y

**Published:** 2025-02-03

**Authors:** Jessica B. Aily, Marcos de Noronha, Ricardo J. Ferrari, Stela M. Mattiello

**Affiliations:** 1 Department of Physical Therapy, Federal University of São Carlos (UFSCar), Rodovia Washington Luís, km 235 - SP-310. São Carlos, São Paulo, CEP 13565-905 Brazil; 2 La Trobe Rural Health School, Rural Department of Allied Health, La Trobe University, PO Box 199, Bendigo, 3552 Victoria Australia; 3 Department of Computing, Federal University of São Carlos (UFSCar), Rodovia Washington Luís, km 235 - SP-310. São Carlos, São Paulo, CEP 13565-905 Brazil

**Keywords:** Intermuscular, Intramuscular, Adipose tissue, Fatty infiltration, MRI, Knee osteoarthritis

## Abstract

**Background:**

People with knee osteoarthritis (OA) may have more thigh intermuscular and intramuscular adipose tissue (interMAT and intraMAT, respectively) compared to those without knee OA. Literature has not considered differences in body mass index (BMI) in the context of comparing intraMAT and interMAT between individuals with and without knee OA, matched for BMI (± 1 kg/m²). This study aims to compare interMAT and intraMAT, along with physical function (including knee extension strength), between individuals with and without knee osteoarthritis, matched by BMI.

**Methods:**

Participants aged ≥ 40 years with symptomatic and radiological knee OA group (grade 2 and 3 on the Kellgren and Lawrence (KL) scale) were included in the affected group, while those with no knee pain and no radiological knee OA changes were included in the unaffected group. No participants were lost to assessment, ensuring complete data analysis for all participants. We used independent t-test and mean difference (95% CI) to compare thigh intraMAT and interMAT volume, self-reported measures (WOMAC questionnaire), physical function measures, and knee extension strength between groups.

**Results:**

Forty-six participants were analyzed (23 in each group). The affected group had significantly higher intraMAT compared to the unaffected group (*p* < 0.05), but no differences were observed for interMAT. Self-reported outcomes and physical function measures were worse in the affected group, as was knee extension strength.

**Conclusion:**

People with knee OA present higher levels of intraMAT and poorer physical function compared to those without knee OA. These findings highlight the need for further research to explore the clinical significance of intraMAT and its potential impact on physical function in this population.

## Background

Knee osteoarthritis (OA) is a prevalent, progressive joint disorder influenced by multiple factors, marked by persistent pain and functional impairment. It represents nearly 80% of the global OA burden and its incidence rises with aging and obesity [1, 2]. Individuals with knee OA often experience persistent pain and significant limitations in daily activities, such as walking, climbing stairs, and transitioning from sitting to standing. These functional impairments are frequently accompanied by declines in muscle strength, particularly in the quadriceps, further exacerbating mobility restrictions and reducing quality of life [3].

Some of the known risk factors for knee OA are older age, female sex, prior joint trauma and obesity [4]. Obesity is considered a mechanical risk factor for knee OA. The mechanism of overloading the knee due to obesity seems to be a very well accepted concept [3]. However, more recently, the consequences of obesity, i.e., the infiltration of adipose tissue intra and inter muscles has been considered a chemical risk factor [5]. A rational for that is drawn from hand OA for example, where overload cannot be considered a factor, yet a region often affected by OA. This leads to the assumption that obesity is not only a mechanical risk factor for OA, but also a chemical factor [6, 7]. Pottie et al. [5] suggests that adipokines, present in obese people, are likely the explanation for the increased risk to develop knee OA from a chemical perspective. In that explanation, the suggestion is that adipokines will synergise with proinflammatory cytokines, leading to the inflammatory symptoms present in not only knee OA but also hand OA [5]. Another factor to consider is level of physical activity, particularly moderate-to-vigorous physical activity. Physical activity is known to influence intramuscular fat infiltration and it is essential to account for physical activity as a potential confounding factor when examining the relationship between knee OA and muscle fat infiltration [8].

In the last few years, some studies have investigated whether the amount of fat tissue and its location in the body would have any particular implication for people with knee OA [9, 10]. In the skeletal muscle, adipose tissue, that is, fatty infiltration, can be divided into two major groups: fatty infiltration within the muscle fibers (intraMAT), and between muscles and beneath fascia (interMAT) [11, 12]. Recently, research has been conducted to investigate whether there are differences in intraMAT and interMAT of the thigh muscles in people with knee OA compared to healthy individuals [12–16]. A meta-analysis published in 2019 with six studies reported that people with knee OA have more interMAT compared to people without knee OA, however for intraMAT, the only study published at that time showed no difference between groups [17]. One important limitation raised by the authors of the meta-analysis was that studies included did not pair groups based on age, height, weight or even body mass index (BMI), raising the possibility that these variables could be confounders and affect the final results [17]. A strong consideration of BMI is essential when designing a study to compare intraMAT and interMAT between individuals with and without knee OA. BMI, while not a direct measure of muscle composition, is associated with overall adiposity and body composition. Higher BMI has been linked to increased fatty infiltration within and between muscles (intraMAT and interMAT) due to systemic effects, such as chronic inflammation and adipokine secretion [5, 12]. Adipokines, which are released in greater quantities in individuals with obesity, interact synergistically with pro-inflammatory cytokines, contributing to fat accumulation in skeletal muscles and systemic low-grade inflammation. This process has been shown to play a role in both metabolic and musculoskeletal disorders, including OA [5, 10]. Failure to account for BMI could introduce confounding effects, as individuals with higher BMI would inherently be expected to have greater muscle fat infiltration regardless of their OA status. Matching groups for BMI, therefore, allows us to isolate the effect of knee OA on muscle fat infiltration. Failure to account for BMI could introduce confounding effects, as individuals with higher BMI would inherently be expected to have greater muscle fat infiltration regardless of their OA status. Matching groups for BMI, therefore, allows us to isolate the effect of knee OA on muscle fat infiltration, enabling more robust comparisons. Previous studies that did not control for BMI reported inconsistent findings regarding intraMAT, further highlighting the need for careful consideration of this variable to reduce bias and improve the validity of conclusions drawn about fatty infiltration in this population.

Therefore, the aim of the current study was to compare the amount of interMAT and intraMAT between a group with knee OA and a comparison healthy group, matched according to sex, age and BMI to avoid the influence of these confounding factors. We also compared physical function between group.

## Methods

### Study design

This was a cross-sectional study to investigate whether there are differences in thigh intramuscular and intermuscular adipose tissue and physical function in individuals with knee OA (affected group) and without knee OA (unaffected group) paired by sex, age and body mass index (BMI). The present study was approved by the local Human Research Ethics Committee of the Federal University of São Carlos (UFSCar), Brazil, under number 3.053.110, in accordance with the Declaration of Helsinki. All eligible participants signed an informed consent after receiving written description of the study and prior to assessments.

### Participants

Participants were recruited from the Sao Carlos (Brazil) community via radio, newspaper, and social media. After expressing interest, participants were interviewed via phone by the principal investigator to verify if they met the initial eligibility criteria.

For both groups, participants had to be sedentary and aged 40 year or over. Sedentary behavior is in fact the norm for people with knee OA, therefore it was a crucial inclusion criterion to minimize variability which are known to influence muscle fat infiltration [8] and to provide accurate representation of the population with knee OA. By standardizing the groups based on sedentary behavior, we aimed to ensure that the observed differences in intraMAT and interMAT were attributable to knee OA rather than differences in physical activity levels. Participants were excluded (from any group) if they were being treated by a physical therapist for any lower limb problem; were walking more than 30 min continuously daily or engaged in regular physical exercises (more than twice a week) up to 6 months prior the study assessments; reported a history of trauma in the lower limbs and/or any previous surgery in their knees; received knee corticosteroid infiltration in the previous 6 months; presented any medical restriction that precluded participation in the study; presented cognitive impairment that compromised the understanding of the tests (obtained by Mini Mental State Examination – MMSE, score of 25 or above considered normal) [18].

Participants met the final specific inclusion criteria for the affected group if they were radiographically diagnosed with unilateral or bilateral knee OA grades II or III according to the Kellgreen and Lawrence criteria (KL) and reported persistent pain, as measured by the WOMAC pain subscale. The WOMAC pain subscale assesses pain during specific functional activities using a Likert scale (0 = none, 4 = extreme) [19]. For inclusion, participants needed a total pain subscale score ≥ 4, which corresponds to the sum of its five items (ranging from 0 to 20), indicating moderate pain intensity during functional activities [20].

Participants met the final specific inclusion criteria for the unaffected group if knee radiography showed grades 0 or 1 on the KL in both knees and no knee pain (WOMAC pain score < 4) [19]. To ensure methodological rigor, we specifically paired participants in both groups based on BMI (± 1 kg/m²), minimizing its potential confounding effect on muscle fat infiltration. This matching process was conducted after initial eligibility screening to ensure that the groups were comparable for BMI, age, and sex, thereby increasing the robustness of our comparisons.

For final eligibility criteria for both groups participants deemed eligible underwent bilateral radiographic exams at the University Hospital (UH) of UFSCar, following standardized imaging protocols [21]. Radiographic diagnoses were conducted by blinded, trained researchers.

Participants also answered the pain subscale of the Western Ontario and McMaster Universities Osteoarthritis Index (WOMAC) [19]. The WOMAC pain subscale is a self-administered questionnaire, translated and validated to Portuguese, consisting of 5 activity questions. Participants were instructed to indicate their level of pain for each activity, using a Likert scale (none, 0; mild, 1; moderate, 2; severe, 3; and extreme, 4). The WOMAC pain score ranges from 0 to 20, with higher scores indicating worse pain intensity [20]. Finally, all participants were also assessed with the MMSE. The MMSE consists of 11 items with a total score of 30 and a participant was considered cognitively impaired if illiterate and total score < 20; or with 1 to 4 years of education and total score < 25; or with 5 to 8 years of education and total score < 26.5; or 9 to 11 years of education and total score < 28; or more than 11 years of education and total score < than 29 [18].

### Data collection

Assessments took place at the Joint Function Analysis Laboratory (LAFAr) and at the Radi-Imagem, a private company experienced in medical imaging exams, in two distinct days, with a time interval between 3 and 7 days. Recruitment and data collection were performed from July 2019 to February 2020. The X-ray was conducted no more than 10 days before the start of data collection to ensure that the imaging reflected the participant’s condition as close as possible to the time of the assessments. The MRI was conducted last, specifically between 3 and 7 days after the physical tests. This sequencing was chosen to avoid any potential influence of physical activity or participant fatigue from the tests on the MRI results, such as muscle composition or structural imaging parameters.

In the first day participants were first submitted to an anthropometric evaluation composed by weight, height and personal information (name, sex and date of birthday), then they answered the WOMAC physical function subscale. After that, participants performed the physical function tests (described below) and the isometric strength test, with the order of assessments randomly decided. All assessments were performed by the same physical therapist/researcher across the study and lasted approximately one hour.

On the second day, participants underwent the thigh Magnetic Resonance Imaging (MRI) scan at Radi-Imagem. To ensure consistency in MRI results, all participants were instructed to refrain from any physical exertion or unusual activities 24 h prior to the MRI scan. This precaution was taken to minimize any transient effects of exercise on muscle water content or tissue signal intensities, which are known to resolve within hours post-exertion [22]. Furthermore, MRI scans were conducted at consistent times of the day (from 6 to 9 am) to account for potential diurnal variations in muscle hydration or metabolic state.

### Outcomes

#### WOMAC physical function subscale

The WOMAC physical function subscale is a questionnaire that individuals complete on their own, which has been translated and validated into Portuguese, containing 17 questions about activities that are answered using a Likert scale (none, 0; mild, 1; moderate, 2; severe, 3; and extreme, 4). The WOMAC physical function score can vary from 0 to 68, with increased scores reflecting poorer function [20].

#### Physical function tests

The functional performance-based tests selected for the present study followed the OARSI recommendations [23]. No warm-up was performed before starting the physical performance tests and participants were able to rest for 2 min between tests.

The 40 m Fast-paced Walk Test (40 m FPWT): participants were instructed to walk as fast as possible without running for a distance of 10 m, returning to the starting point and repeating the walk for a total distance of 40 m. Running and the use of walking aids was not allowed at any point during the test. The test was conducted in a flat, well-lit indoor corridor with a smooth, non-slippery surface to ensure participant safety. Participants were instructed to wear comfortable, flat-soled shoes during the test. To further ensure safety, a researcher was present to provide any assistance if needed. The test was timed and the speed in meters/second (m/s) was used for analysis [23, 24].

The 30-second Chair Stand Test (30s-CST): from the sitting position, with feet flat on the floor and arms crossed over the chest, participants were instructed to stand up completely from the chair and then sit down as quickly as possible for 30s, without using their hands. The total number of repetitions performed was recorded [23, 25].

The Stair Climb Test (12-step SCT): participants were instructed to climb a flight of 12 regular steps (16 cm high each) and immediately descend as quickly as possible safely, using the handrail only if needed. The total test duration was timed (in seconds) [23, 25].

A “failed test” was predefined in the study protocol as the inability to complete the task within the allocated time frame or in adherence to the standardized instructions. Notably, all participants successfully completed the physical performance tests, and no instances of failed tests were observed or included in the analysis.

#### Knee extension strength

The maximal knee extension isometric peak torque was measured using an isokinetic dynamometer (Multi-Joint System 3, Biodex Medical System, New York, USA) following the protocol by Serrao et al. [26]. In short, the assessments were conducted with participants seated on the device chair, with knees flexed at 60^o^, body stabilized by straps, and arms crossed in front of the chest. Participants performed 3 submaximal isometric contractions in order to become familiar with the test procedure. After familiarization, participants were instructed to rest for 2 min. The isometric test consisted of three maximal contractions at 60^o^ of flexion held for 3 s each, with a 1-min rest between contractions [26–28]. Verbal encouragement was given during all contractions.

Isometric peak torque was calculated as the average of the three maximal contractions [29]. For statistical analysis, both the non-normalized isometric peak torque (Nm) and the normalized isometric peak torque (peak torque/body mass * 100) were used to provide a comprehensive assessment of knee extension strength.

#### InterMAT and IntraMAT

Thigh interMAT and intraMAT were assessed using 3-D images acquired from a 1.5 T GE Signa LX Goldseal MR Scanner (General Electric, Milwaukee, WI, USA). Participants were placed in supine, and images of the thigh were acquired using a body coil. The mid-thigh was defined according to a vitamin E tablet attached at the middle point between the greater trochanter and the lateral condyle of the femur. T1-weighted, spin-echo sequence images were acquired with the following parameters: plane of acquisition = axial; repetition time = 1280 ms; echo time = 22.6 ms, optimized field of view = 512 ⨉ 512 mm; slice thickness = 3 mm; slice gap = 0 mm; and number of slices = 24. All participants were instructed to remain as still as possible during image acquisition. Image acquisition was performed by a 15-years experienced radiologist who works for the company.

**Fig. 1:**
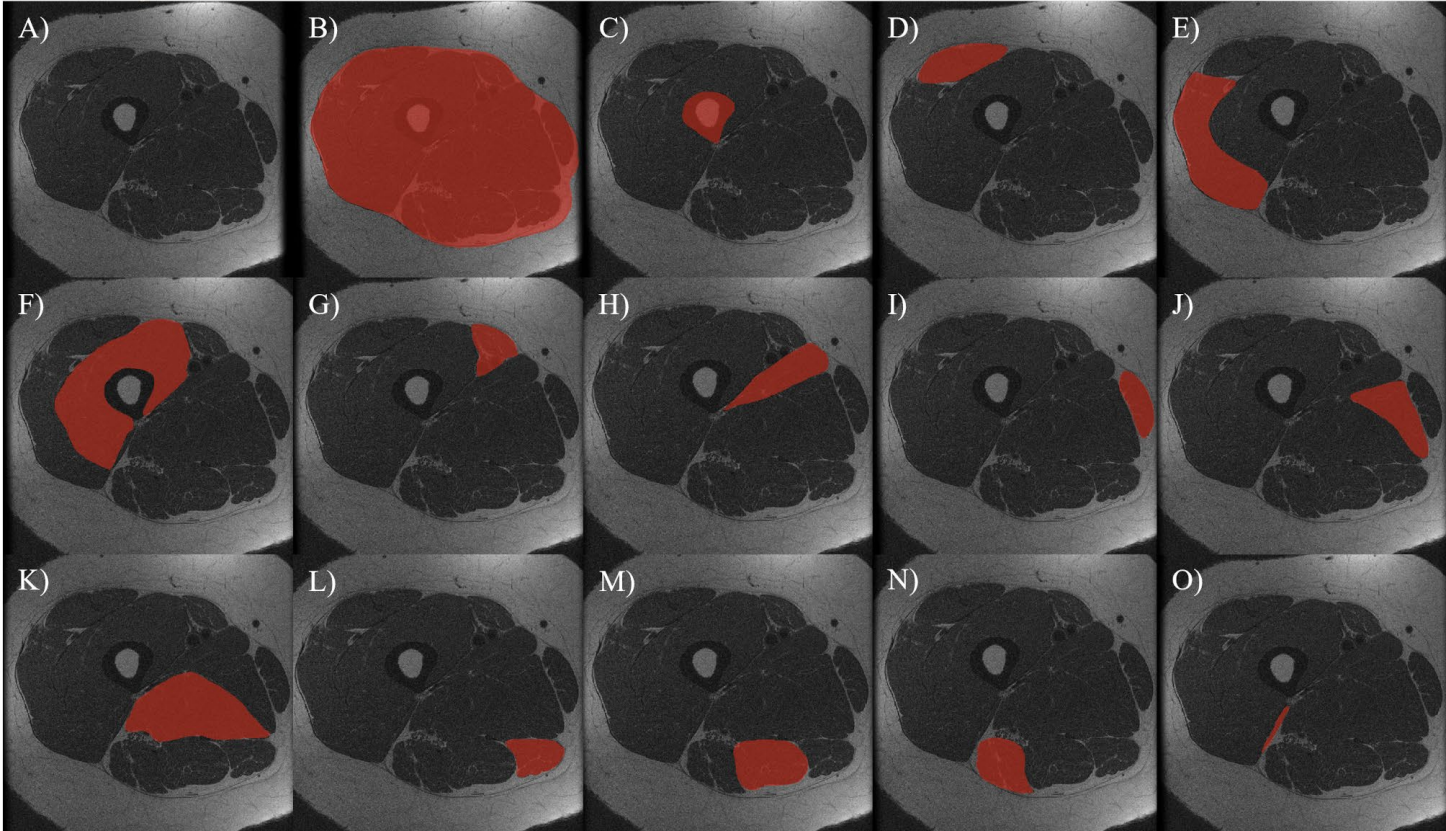
Manual segmentation of all thigh muscles and bone. (**A**) Original image; (**B**) Muscular fascia; (**C**) Thigh bone (femur); (**D**) Rectus Femoris Muscle; (**E**) Vastus Lateralis Muscle; (**F**) Vastus Intermedius Muscle and Vastus Medialis Muscle; (**G**) Sartorius Muscle; (**H**) Adductor Longus Muscle; (**I**) Gracilis Muscle; (**J**) Adductor Brevis Muscle; (**K**) Adductor Magnus Muscle; (**L**) Semimembranosus Muscle; (**M**) Semitendinosus Muscle; (**N**) Long Head Biceps Muscle; (**O**) Short Head Biceps Muscle

One trained, experienced segmenter (JBA) analyzed all MRI scans manually using the ITK-SNAP (version 3.6) software, as demonstrated in Fig. 1. The ten middle slices were selected for segmentation, representing a 30 mm region of interest. This number of MRI slices was found to provide an accurate estimate of total extensor muscle volume in the lower leg [30]. The segmenter was blinded to group allocation.

#### MRI image segmentation and tissue volume calculations

Because of the relatively large slice thickness, in this study we processed each slice individually and the results were combined to provide the tissue volumes. The image processing starts by preprocessing all MRI images with the Non-Local Means technique [31] for noise reduction, followed by the N4-ITK technique [32] for bias field correction and normalization of the voxel intensity range of all images to the [0; 255] range. Then, by combining the manual segmentation of all thigh muscles and bone, we automatically created binary masks representing the intraMAT, interMAT and fascia regions. Next, we segmented the intraMAT and interMAT tissues in the MRI image using an adaptive thresholding, which locally computes a threshold value as the weighted Gaussian mean of the local neighborhood pixels. In this study, the size of the local region was defined as 55 pixels since it provided the segmentation results that best correlate to visual analysis of a segmenter expert (JBA). After that, we used the intraMAT and interMAT binary masks to define the regions to count the number of pixels corresponding to each tissue. Finally, we used the image pixel size to define the volume of the segmented regions and normalize their values by the fascia volume. All volumes were expressed as cm^3^. Again, the researcher responsible for all calculations was blinded to group allocation.

### Statistical analysis

Sample size was calculated based on a pilot dataset with ten participants in two groups, with similar characteristics to those of the current study (age, BMI, sex and OA severity), using the software G*Power, version 3.1. A significance level of 0.05 and power of 0.80 were considered to detect an intraMAT difference between groups of at least 3.0 cm^3^ (SD = 3.5 cm^3^). Thus, 23 participants were required for each group.

All analyses were performed using the Statistical Package for Social Science, version 20.0 (SPSS Inc, Chicago, IL, USA). Normal distribution of data was assessed using Kolmogorov-Smirnov test and skewness values, where values inside the range − 1.0 to 1.0 indicate normal distribution [33]. This method is appropriate for the sample size and has been widely used in similar studies. Independent t-test was used to compare all variables between groups (Age, BMI, WOMAC, physical function tests, isometric torque, intraMAT, interMAT). Mean, standard deviation, mean difference between groups and 95% confidence interval and Cohen’s effect size for the differences were also calculated.

## Results

**Fig. 2:**
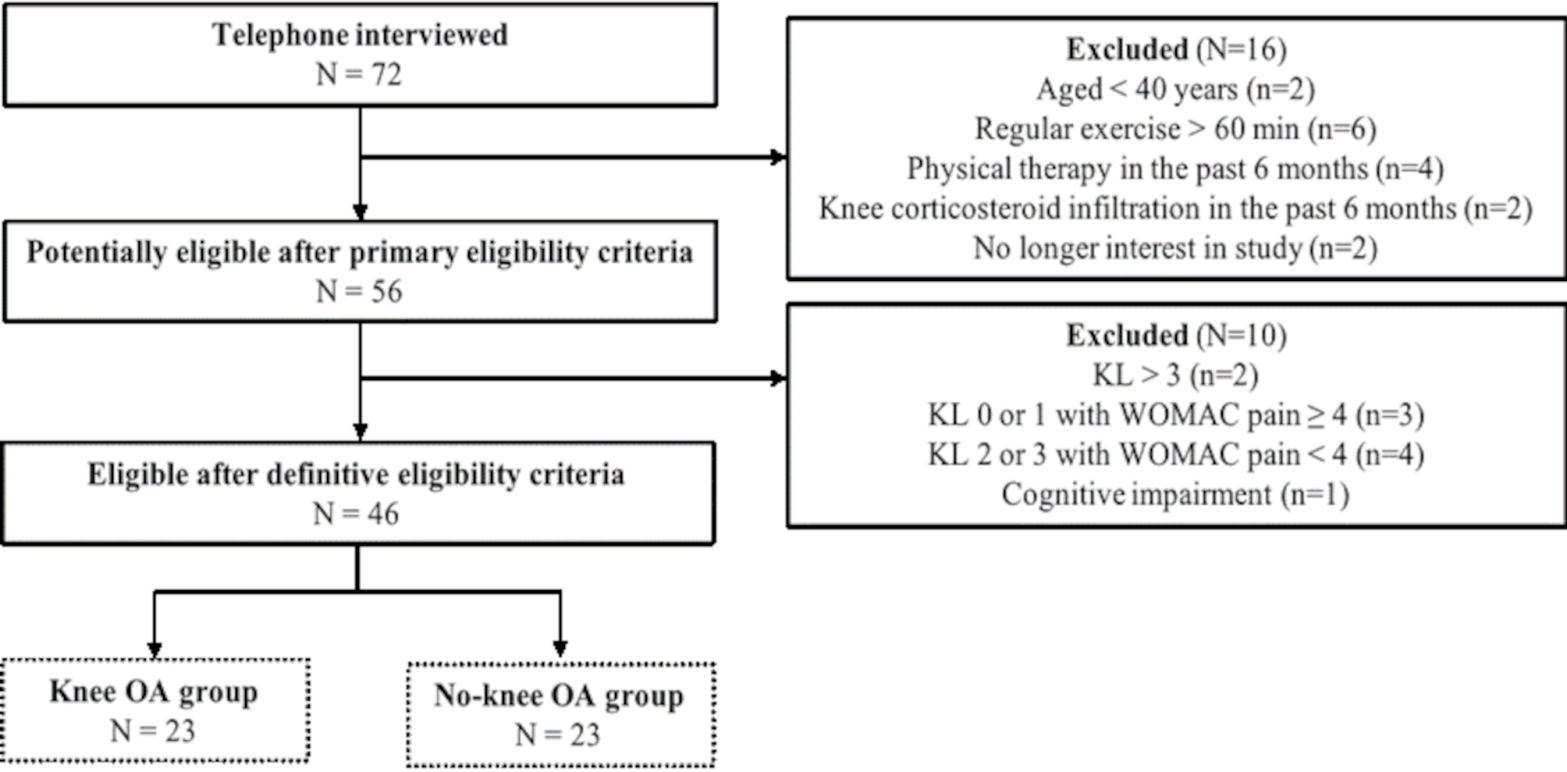
Flow Diagram for Participant Recruitment and Grouping

Seventy-two potential participants expressed interest in the study and were contacted by the principal investigator. From these, 56 who met the initial eligibility criteria were further screened, and 46 met the final criteria and were included in the study (23 diagnosed with knee OA and 23 without knee OA; Fig. 2). Each group consisted of 12 women and 11 men. In the affected group, 10 participants had bilateral knee OA, and 13 had unilateral. Regarding knee OA severity, 12 participants were classified as grade 2, and 11 as grade 3 according to the Kellgren and Lawrence criteria. All participants provided consent and signed the informed consent form. Characteristics of participants are presented in Table 1. Successful pairing of groups was confirmed as there was no difference between groups for age and BMI. Details of the BMI matching process are outlined in the Methods section. The expected differences in function, performance, and pain between groups were statistically significant (Table 1; *p* > 0.05).

**Table 1:** Characteristics of participants and between group comparison for thigh volume for intermuscular adipose tissue (interMAT) and intramuscular adipose tissue (intraMAT), self-reported physical function (WOMAC questionnaire), physical function measures and knee extensors strength

	Affected group	Unaffected group	*p* value	Mean difference	95% Confidence Interval	Cohen’s d
	(*n* = 23)	(*n* = 23)				
Age (y)	61.9 ± 9.5	62.0 ± 9.8	0.963	0.1	-5.7 to 5.4	0.01
BMI (kg/m^2^)	27.0 ± 2.5	26.7 ± 1.6	0.600	0.3	-0.9 to 1.6	0.14
WOMAC						
Pain (score)	8.0 ± 3.3*	0.1 ± 0.3	< 0.001	7.9	6.5 to 9.3	3.37
Function (score)	26.6 ± 11.7*	1.8 ± 1.5	< 0.001	25.5	20.5 to 30.5	2.97
Physical function tests						
30s-CST (reps)	9.9 ± 2.7*	14.0 ± 2.2	< 0.001	4.1	2.7 to 5.6	1.66
40 m FPWT (m/s)	1.6 ± 0.3*	1.9 ± 0.3	< 0.001	0.4	0.2 to 0.5	1.00
12-step SCT (s)	17.5 ± 11.1*	10.8 ± 2.1	0.010	6.6	1.9 to 11.4	0.84
Isometric torque (Nm/kg x 100)	157.9 ± 45.1*	206.0 ± 40.8	< 0.001	48.1	22.5 to 73.6	1.12
Isometric torque (Nm)	118.8 ± 44.5	141.4 ± 37.8	0.070	22.6	-47.1 to 1.95	0.55
InterMAT (cm^3^)	21.7 ± 9.9	18.7 ± 10.6	0.337	2.9	-3.2 to 9.0	0.29
IntraMAT (cm^3^)	9.9 ± 5.0*	6.2 ± 3.5	0.005	3.7	1.2 to 6.3	0.83

Values are expressed as mean ± SD

Abbreviations: BMI, body mass index; WOMAC, Western Ontario and McMaster Universities Osteoarthritis Index; y, years; s, seconds, Nm, newton-metre; kg, kilogram; cm, centimeter; reps, repetitions; 40 m FPWT, 40 m Fast-paced Walk Test; 30s-CST, 30-second Chair Stand Test; 12-step SCT, Stair Climb Test

(*) *p* < 0.05, significantly different compared with the unaffected group

There was no between group difference for interMAT, however, the affected group had higher levels of IntraMAT compared to the unaffected group (*p* < 0.05; Table 1).

For physical function, outcomes assessed included the 30s-CST, 40 m FPWT, 12-step SCT, and isometric peak torque. These measures indicated poorer physical function in the affected group compared to the unaffected group (Table 1). For patient-reported outcomes, the affected group reported worse scores on the WOMAC function subscale. These findings align with the clinical profile of knee OA.

## Discussion

This is the first study comparing thigh interMAT and intraMAT between people with and without knee OA, matched specifically for sex, age and BMI. Our main findings are that people with knee OA have more thigh IntraMAT when compared to people without knee OA, and there was no difference between groups for interMAT.

Current literature comparing thigh intraMAT between people with and without knee OA is very limited. Kumar et al. [12] reported that people with knee OA had more quadriceps intraMAT fraction compared to people without knee OA, but this difference was not observed when analyzing other thigh muscles or all thigh muscles combined. Furthermore, when intraMAT volume (as opposed to fraction) was assessed, no differences between groups were found. The similar outcome analysed in the study by Kumar et al. [12] and the current study was the intraMAT for all thigh muscles, and the results are conflicting.

One possible explanation for these conflicting results is the importance of matching the groups. Kumar et al. [12] adjusted their analysis for age, sex and BMI, but did not match groups at recruitment, which may have limited their ability to detect significant differences. In contrast, our study used specific criteria to ensure that the groups were comparable regarding age, sex and BMI. As a result, we found a significant difference in intraMAT volume between groups (Table 1), highlighting the importance of matching groups.

It is important to note that knee OA can lead to reduced physical activity due to pain and functional limitations, which may, in turn, contribute to increased intraMAT [34]. This suggests a bidirectional relationship, where OA promotes fat infiltration, and increased fat infiltration may further impair muscle quality and physical function [12, 34, 35]. Understanding this relationship is critical, as higher intraMAT has been associated with reduced muscle strength and functional performance in people with knee OA [12, 34, 35].

We are aware of one other study that compared intraMAT between people with and without knee OA, this time only focusing on vastus medialis [36]. Similar to Kumar at al. [12], Teoli et al. [36] adjusted their analysis based on age, sex and BMI but did not match groups at recruitment, reporting no difference in intraMAT volume between groups. This further highlights the importance of recruiting matched groups to control for potential confounding factors. Our findings reinforce that careful matching for age, sex, and BMI is essential to isolate the role of knee OA in muscle fat infiltration and provide more robust conclusions.

For interMAT, literature has a larger number of studies comparing people with and without knee OA. A systematic review by Pedroso et al. [17] comparing fatty infiltration in the thigh muscles of people with knee osteoarthritis presented a meta-analysis specifically for thigh interMAT, reporting that people with knee OA had more interMAT when compared to people without knee OA. In contrast, the present study found no difference between groups for interMAT. However, the results by Pedroso et al. [17] is the pooled result of 6 studies, which individually, the 3 largest studies reported a difference between groups [13, 14, 37]. These 3 particular studies had 504, 125 and 86 participants, respectively [13, 14, 37], which seems to be an indication that the difference between groups, although present, is small, therefore a study without an adequate sample size for this particular analysis would likely present a type 2 error. This possibility cannot be discarded in our study as our sample size of 46 participants is similar to the studies in the meta-analysis by Pedroso et al. [17] that individually did not find a difference between groups [12, 15, 16].

The clinical implications of greater intraMAT in individuals with knee OA warrant further investigation. Studies have shown that increased intraMAT in the vastus medialis (VM) muscle is associated with worsening cartilage damage, poorer functional abilities, and worsening symptoms [28, 35, 38, 39]. Notably, intraMAT appears to increase earlier than muscle atrophy [28, 40], suggesting its potential role as an early indicator of muscle degeneration in knee OA. IntraMAT, which reflects fat infiltration within muscle fibers, may have a more detrimental effect on muscle quality and strength compared to interMAT, further exacerbating functional impairments in knee OA. These findings highlight the need for future studies to explore intraMAT as a biomarker for disease severity and a target for interventions, such as physical activity or resistance training programs. Longitudinal studies are particularly needed to evaluate whether reducing intraMAT improves muscle function, pain, and disease outcomes.

Regarding muscle strength, physical function tests and WOMAC, not surprisingly our results confirm previous studies showing that people with knee OA have less function and strength than people without knee OA [3, 41]. To a degree, the purpose of these tests was to ensure that our cohort of people with knee OA are similar to the population of other studies, which was confirmed by the results of these assessments (Table 1).

While our study had the strength of being the first study comparing intraMAT and interMAT between people with knee OA and matched individuals without knee OA (by BMI, age and sex), we can acknowledge some limitations. The first is perhaps the small sample size of the study, particularly to identify difference between groups in interMAT. A larger sample size would give us more confidence that our results are not type 2 error. Another limitation noted in our study is limited likelihood for these assessments to be performed clinically. Assessment of fatty infiltration in thigh muscles proved to be costly and time consuming, making it inviable for it to be measured on a day-to-day basis. Future studies could consider the investigation of cheaper methods to collect data on fatty muscle infiltrations along with the development of automation of the process to measure the amount of intraMAT and interMAT. Also, other approaches for the several analyses could have been considered. For example it seems reasonable to analyse average isometric peak force after normalizing it by quadriceps cross-sectional area or volume, however we did not have these data available and therefore it is something to be considered in the future. The lack of control for painkiller could also have generated a larger difference between groups for function and strength. Lastly, clinicians would benefit from future studies investigating whether intraMAT and interMAT can be modified in people with knee OA, and whether that possible change would affect clinical outcomes such as pain and function.

## Conclusion

Apart from reinforcing the notion that people with knee OA have less function, our results showed that people with knee OA have more intraMAT around the thigh than individuals without knee OA matched by BMI, age and sex. That same difference was not found for interMAT however this could have been a type 2 error.

## Data Availability

The datasets used and/or analysed during the current study are available from the corresponding author on reasonable request.

## Abbreviations

OA: Osteoarthritis
IntraMAT: Fatty infiltration within the muscle fibers
InterMAT: Fatty infiltration between muscles and beneath
BMI: Body mass index
MMSE: Mini Mental State Examination
KL: Kellgreen and Lawrence criteria
WOMAC: Western Ontario and McMaster Universities Osteoarthritis Index
LAFAr: Joint Function Analysis Laboratory
MRI: Magnetic Resonance Imaging
